# Primitive reflexes as candidate quantitative readouts of hierarchical inhibitory control across the lifespan

**DOI:** 10.3389/fnhum.2026.1860053

**Published:** 2026-06-02

**Authors:** Erzsébet Stephens-Sarlós, Attila Szabo

**Affiliations:** Faculty of Health and Sport Sciences, Széchenyi István University, Győr, Hungary

**Keywords:** cortical inhibition, developmental neuroscience, GABA, inhibitory control, neuroplasticity, primitive reflexes, sensorimotor integration

## Abstract

Primitive reflexes are typically inhibited during normal neurodevelopment as cortical and subcortical inhibitory systems mature. However, persistent or incompletely integrated primitive reflexes have been observed in neurodevelopmental conditions including attention-deficit/hyperactivity disorder (ADHD), dyslexia, developmental coordination difficulties, sensory-processing-related presentations, and delayed speech and language development. Re-emergence of primitive reflexes has also been reported in aging and neurological disorders such as Alzheimer’s disease and Parkinson’s disease. Based on these observations, this paper explores the hypothesis that primitive reflex expression may reflect distributed hierarchical inhibitory systems spanning spinal, brainstem, and cortical levels. We propose that persistence or reappearance of early stereotyped reflex patterns may indicate altered inhibitory regulation and sensorimotor integration associated with changes in GABAergic function and activity-dependent neural plasticity. We further propose that quantitative reflex profiling may represent a candidate behavioral framework for studying distributed inhibitory regulation across the lifespan. Reflex integration patterns may relate to motor control, sensory processing, attentional regulation, and broader neurocognitive functioning. However, clinical applicability depends on standardized measurement procedures, validated protocols, multimodal neurophysiological correlations, and replication. The proposed model should currently be interpreted as a theoretical and hypothesis-generating framework rather than a validated mechanistic or clinical account. Primitive reflex profiling is not yet part of standard clinical or neurophysiological assessment. Future research should prioritize longitudinal, multimodal, and interventional studies testing relationships between primitive reflex expression, inhibitory network organization, cortical dynamics, and sensorimotor function. Primitive reflexes may therefore represent developmentally informative behavioral phenomena associated with distributed inhibitory systems across the lifespan.

## Introduction

This Perspective is motivated by recurring observations of persistent primitive reflex expression across developmental and later-life contexts. Primitive reflexes are generally expected to become progressively attenuated during neurodevelopment as hierarchical inhibitory regulation and cortical top-down modulation mature. However, persistent or re-emergent reflex expression has repeatedly been reported in both neurodevelopmental and neurodegenerative conditions, suggesting that primitive reflexes may reflect broader properties of distributed sensorimotor and inhibitory system organization rather than merely transient neonatal phenomena (Matuszkiewicz and Galkowski, 2021; Pecuch et al., 2020; Schott and Rossor, 2003; Sigafoos et al., 2021; Zafeiriou, 2004). At the same time, primitive reflex expression is unlikely to reflect inhibitory control alone and may additionally be influenced by muscle tone, posture, sensory processing, attentional state, biomechanical constraints, arousal, and examiner-dependent factors. Accordingly, the present framework should be interpreted as one possible explanatory model rather than a definitive account of primitive reflex persistence.

This viewpoint is informed by preliminary observations across two distinct developmental periods. In school-age children, we examined primitive reflex profiles in relation to sensorimotor development and auditory–visual functions, and compared reflex persistence before and after a structured Sensorimotor Training Program (STP) (Stephens-Sarlós et al., 2024a). The STP is a personalized sensorimotor intervention framework designed to target persistent primitive reflexes through reflex-specific movement patterns and individualized bottom-up and top-down exercises. In older adults, a 16-week sensorimotor intervention was associated with reductions in primitive reflex expression together with changes in cognitive and mental-health-related measures, consistent with the possibility that reflex expression may remain modifiable across the lifespan through mechanisms related to neural plasticity (Stephens-Sarlós et al., 2024b). These observations do not establish causality, but they motivate the hypothesis that primitive reflexes may serve as accessible behavioral indicators of distributed inhibitory and sensorimotor regulation.

Accordingly, we aim to clarify why primitive reflexes may serve as potential quantitative readouts of distributed inhibition, why they may serve as clinically relevant indicators following the expected developmental window of cortical modulation, and how bottom-up and top-down practice modalities can be formulated into empirically testable mechanistic predictions and intervention tests.

### Measurement infrastructure

A quantitative readout framework requires standardized measurement. We developed a structured assessment framework covering 18 primitive reflexes (the validation manuscript is currently under peer review) to support systematic reflex evaluation. The assessment battery includes oral reflexes (e.g., rooting, sucking), grasp-related reflexes (e.g., palmar and plantar grasp), tonic reflexes (e.g., ATNR, STNR, TLR), protective reflexes (e.g., Moro, diving, fear paralysis), and transitional postural reactions commonly assessed in developmental neurology. Reflex expression is evaluated using a three-point ordinal scoring system with predefined elicitation and response criteria, and preliminary analyses indicate high inter-rater and intra-rater reliability. At present, the framework should be interpreted as a structured research-oriented assessment approach rather than a clinically validated biomarker platform.

However, full documentation of the scoring structure, validation procedures, and comprehensive psychometric evaluation is reported separately, and the present framework should be considered an ongoing methodological development rather than an established measurement infrastructure.

Ongoing studies, including fNIRS-based approaches, aim to operationalize the relationship between persistent reflex expression and cortical inhibitory dynamics. This Perspective provides a conceptual and experimental framework for these efforts. These platforms could enable hypothesis-driven testing of relationships between reflex expression, cortical activation, behavioral variability, and intervention-related change.

### Primitive reflexes as embodied brainstem–spinal programs

Primitive reflexes are early-emerging, evolutionarily conserved, stereotyped sensorimotor responses elicited by specific sensory stimuli during early development. Examples include the rooting reflex, sucking reflex, Moro reflex, palmar grasp reflex, and asymmetric tonic neck reflex (ATNR). These responses are implemented primarily through brainstem and spinal circuits that transform sensory input into relatively stereotyped motor output, and they progressively come under cortical inhibitory modulation as the nervous system matures (Futagi et al., 2012; Walker et al., 1990; Schott and Rossor, 2003; Zafeiriou, 2004). Rather than functioning as isolated motor events, primitive reflexes are embedded within the infant’s whole-body sensorimotor state, where muscle tone, posture, arousal, and biomechanical constraints can shape their amplitude, symmetry, and coordination.

In this context, primitive reflexes can be understood as motor primitives whose execution depends on the organism’s biomechanical and neuromuscular state. In the neonate, global muscle tone strongly shapes reflex amplitude and coordination: hypotonia in preterm or neurologically immature infants is associated with attenuated or poorly coordinated reflexes, whereas abnormal hypertonia can exaggerate reflex expression or delay integration (Mercuri et al., 2003; Zafeiriou, 2004). Primitive reflexes therefore emerge from interactions between central pattern–generating circuits and tonic postural systems, mediated by vestibulospinal and reticulospinal pathways, and are embedded within a whole-body sensorimotor context (Takakusaki et al., 2024).

Primitive reflexes are thought to serve an adaptive function within the early sensorimotor environment and may also have developmental and clinical significance. Rooting and sucking reflexes support feeding and airway coordination, whereas startle and withdrawal reflexes provide rapid protective responses. Tonic reflexes couple head position to limb tone and contribute to the initial organization of posture and orientation (Zafeiriou, 2004). These functions scaffold the transition from reflexive to voluntary action and position primitive reflexes as active components of developmental organization rather than passive remnants of immaturity. Contemporary developmental motor frameworks further emphasize that early motor behavior progressively evolves toward adaptive variability, flexibility, and context-dependent control through ongoing sensorimotor experience and neural maturation (Hadders-Algra, 2018).

Developmental integration of primitive reflexes is commonly attributed to the maturation of descending inhibitory control. Clinically, persistent reflexes in childhood mark neurological immaturity, whereas their reappearance in older adults signals reduced cortical restraint and is associated with dementia and diffuse cerebral dysfunction (Damasceno et al., 2005; Gabelle and Gutierrez, 2016; Harp et al., 2022; Nicolson and Fawcett, 2005; Plutino et al., 2019; Rao, 2000; Zafeiriou, 2004).

Although often described as cortical “suppression,” integration is more accurately conceptualized as a reconfiguration of distributed control architectures spanning spinal, brainstem, and cortical levels. In this framework, primitive reflexes are observable outputs of hierarchical inhibitory control within a sensorimotor system, and their expression mirrors the balance of excitation and inhibition across multiple scales (Luu et al., 2024).

### GABAergic maturation and inhibitory architecture

A central neurochemical substrate of hierarchical inhibitory control is the maturation of GABAergic signaling. During early development, gamma-aminobutyric acid (GABA) can exert depolarizing effects before progressively shifting toward predominantly inhibitory signaling as chloride homeostasis and inhibitory circuit organization mature, reorganizing network excitability and enabling selective gating of sensorimotor loops (Ben-Ari, 2002; Cossart and Khazipov, 2022). More recent frameworks emphasize that inhibitory maturation reflects a distributed, activity-dependent developmental process involving network stabilization, synaptic refinement, and experience-dependent regulation of cortical dynamics rather than a single discrete developmental switch (Wen and Turrigiano, 2024; Manley et al., 2024).

Experimental animal model evidence suggests an association between reflex attenuation and the maturation of spinal inhibitory circuitry. During postnatal grasp development, increased presynaptic GABAergic inhibition of sensory inputs to spinal interneurons accompanies reflex suppression, demonstrating that reflex integration reflects distributed strengthening of inhibitory circuits across spinal and supraspinal circuits (Laliberte et al., 2022). Across the lifespan, cortical GABA follows a nonlinear trajectory—increasing during development, stabilizing in early adulthood, and declining with age (Porges et al., 2021). Persistent primitive reflexes in childhood and their re-emergence in later life may be consistent with a shared inhibitory substrate linking neurodevelopmental immaturity and neurodegenerative decline, although this relationship remains indirect and may be influenced by multiple interacting factors (Damasceno et al., 2005; Porges et al., 2021).

This Perspective reframes primitive reflexes as behavioral readouts of inhibitory architecture. Primitive reflexes are not necessarily abolished during development; instead, their observable expression becomes progressively attenuated through processes of inhibitory control and integration within distributed neural circuits. Their persistence may reflect incomplete maturation of this architecture, although alternative explanations—including differences in muscle tone, postural control, arousal, sensory processing, attention, biomechanical constraints, and examiner-dependent factors—should also be considered, whereas their reappearance may reflect its degradation.

### Movement-dependent modulation of inhibitory control

Inhibitory architecture is dynamically modulated by movement. Acute physical activity increases cortical GABA concentrations as measured by magnetic resonance spectroscopy, and systematic review evidence confirms post-exercise elevations in GABA and related metabolites (Coxon et al., 2014; Maddock et al., 2016; Ryberg et al., 2023). Recent syntheses propose exercise as a mechanism for preserving inhibitory integrity in later life by supporting GABAergic function and network stability (Novak et al., 2024).

Exercise-related effects may additionally involve anti-inflammatory processes, metabolic signaling, gut–brain interactions, and exercise-induced peripheral mediators such as exerkines and myokines (Ribeiro et al., 2022).

Beyond neurochemistry, human meta-analytic evidence indicates that exercise is associated with increased peripheral BDNF levels, a plausible mediator of exercise-related cognitive and affective benefits (Szuhany et al., 2015).

Experimental animal and early developmental studies suggest that BDNF may contribute to the maturation of inhibitory circuitry during postnatal development, providing a potential mechanistic link between neurotrophic signaling and inhibitory circuit refinement (Huang et al., 1999; Park and Poo, 2013).

Activity-dependent myelination has been proposed as an additional channel through which repeated sensorimotor engagement may stabilize circuit communication and support more efficient hierarchical gating (Fields, 2015).

Beyond conduction efficiency, activity-dependent myelination may contribute to the temporal coordination of coincident neural activity and spike timing-dependent plasticity within distributed cortical networks, processes implicated in neurodevelopmental and psychiatric disorders, including ASD and schizophrenia (Pajevic et al., 2014; Andrade-Talavera et al., 2023).

Although infant-specific data remain limited, the available findings collectively suggest a plausible, yet still hypothetical, developmental model in which spontaneous and structured movements may bias emerging neural circuits toward enhanced inhibitory regulation (Adolph and Hoch, 2019).

Foundational mechanistic insights into developmental reflex organization and inhibitory maturation have also emerged from animal models, particularly rodent spinal and sensorimotor circuit studies, which continue to provide experimentally tractable systems for investigating causal mechanisms (Smith et al., 2017; Nguyen et al., 2017).

Movement may function as a putative endogenous modulator of inhibitory maturation, potentially through repeated engagement of sensorimotor loops and activity-dependent plasticity. More broadly, developmental evidence suggests that motor activity can shape higher-order cognitive and neural organization across multiple developmental domains (Iverson, 2010); however, the extent to which these mechanisms directly contribute to primitive reflex attenuation remains to be empirically established.

This framework motivates a central unresolved mechanistic question: whether repeated execution of primitive reflex motor patterns, or the deliberate performance of voluntary movements that oppose those patterns, may contribute to inhibitory control over persistent primitive reflexes through activity-dependent cortical and subcortical plasticity. Preliminary work has begun to examine movement-dependent modulation of reflex expression, suggesting that structured sensorimotor activity may be associated with changes in inhibitory balance, although causal relationships remain to be established. Clarifying this relationship is essential for distinguishing correlation from causation in the integration of primitive reflexes (Stephens-Sarlós et al., 2024a,b, 2025).

We suggest decomposing sensorimotor training into two opposing intervention axes: bottom-up (peripheral-to-spinal/brainstem) training emphasizing sensory gain and postural tone, and top-down (cortical-to-brainstem/spinal) training emphasizing context-dependent suppression, executive control, and motor learning (Coxon et al., 2014; Seki et al., 2003).

**Figure 1:**
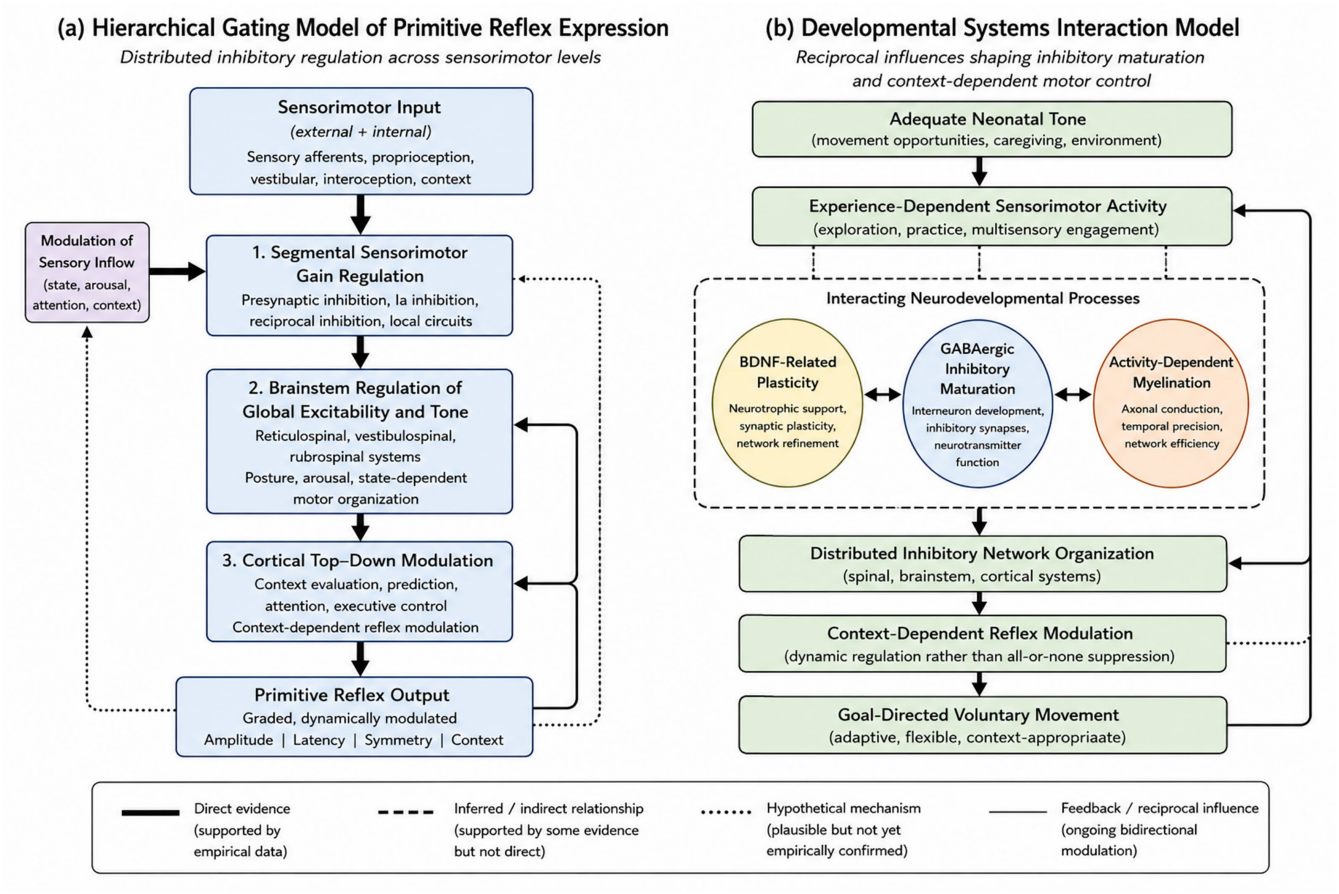
Hypothesized hierarchical gating framework and developmental systems cascade of primitive reflex expression. **(a)** Hierarchical gating model. Primitive reflex expression is conceptualized as the emergent outcome of distributed inhibitory regulation across interacting spinal, brainstem, and cortical levels of the sensorimotor system. Spinal gain control mechanisms, including presynaptic inhibition and modulation of sensory inflow, shape reflex responsiveness at the segmental level. Brainstem-mediated regulation of global excitability and muscle tone involves reticulospinal and vestibulospinal pathways that contribute to posture, arousal, and state-dependent motor organization. Cortical top-down systems further contribute context-dependent modulation and suppression of reflex-associated motor output. Within this framework, primitive reflexes are treated as graded and dynamically modulated phenomena rather than all-or-none responses. **(b)** Developmental systems cascade (interaction model). A systems-level developmental sequence is hypothesized in which early sensorimotor activity interacts with multiple parallel neurobiological processes, including BDNF-related plasticity, maturation of GABAergic inhibitory circuitry, and activity-dependent myelination. These interacting processes are proposed to contribute to the progressive organization of distributed inhibitory networks and the emergence of increasingly context-dependent voluntary motor control. Importantly, the model emphasizes reciprocal influences, ongoing feedback loops, and modulatory effects from contextual and developmental factors rather than a single deterministic developmental pathway. Although several individual components are supported to varying degrees by existing literature (e.g., activity-dependent plasticity, inhibitory maturation, and myelination), the integrated developmental sequence and its causal structure remain to be empirically tested using longitudinal, multimodal, and interventional research designs.

**Figure 2:**
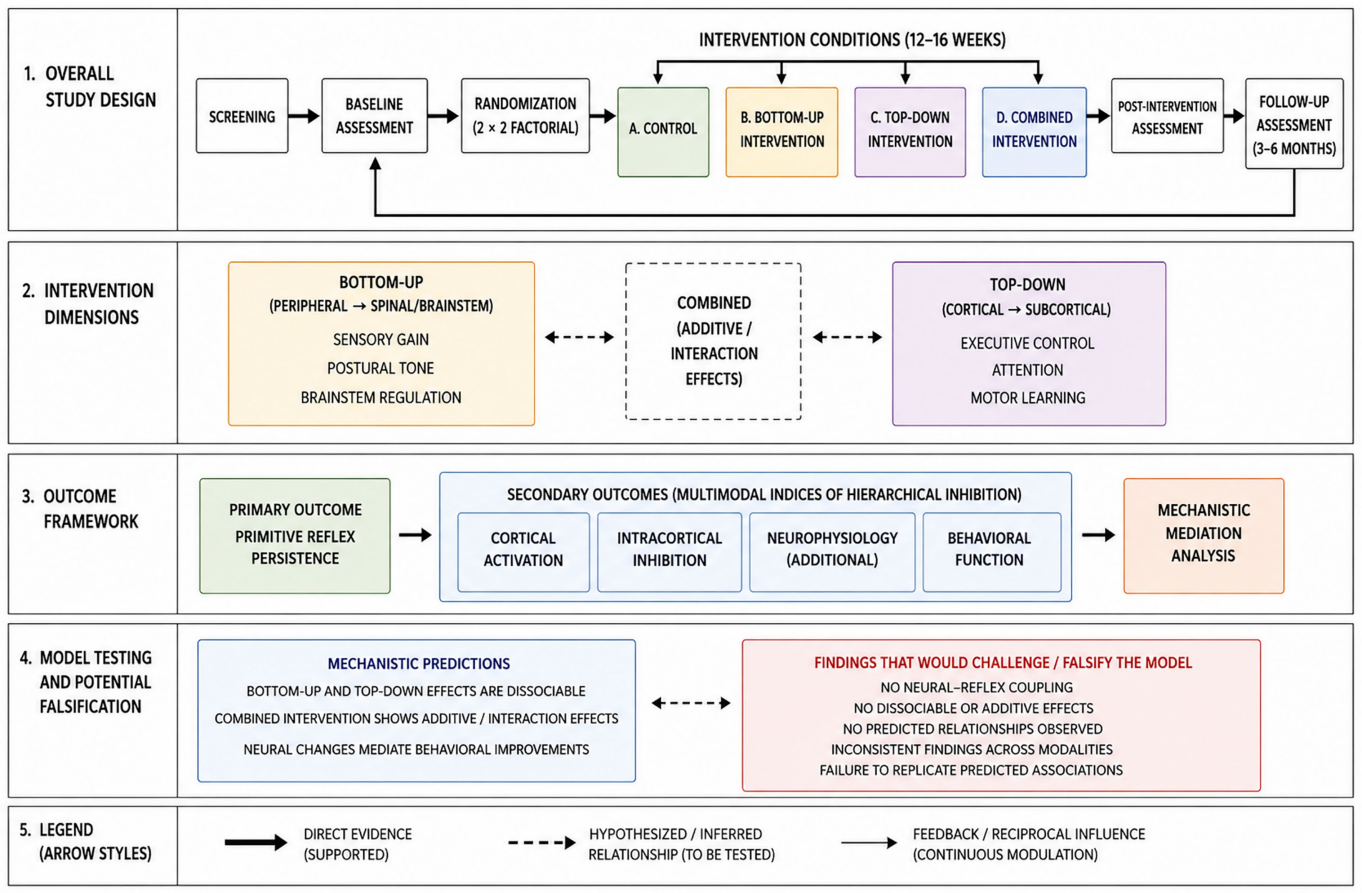
Hypothesized factorial experimental framework for evaluating the hierarchical gating model of primitive reflex expression.

The hierarchical gating framework and the developmental cascade hypothesis are summarized in Figure 1, and the intervention logic in Figure 2.

Figure 1 illustrates a proposed, hypothesis-generating conceptual framework and should not be interpreted as an established causal pathway. The depicted relationships integrate direct evidence, inferred developmental associations, and testable mechanistic hypotheses. Arrow styles indicate different evidential levels: solid arrows represent relatively direct evidence, dashed arrows represent inferred or indirect relationships, and dotted arrows represent hypothetical or currently untested mechanisms.

Figure 2 outlines a proposed experimental framework designed to evaluate mechanistic predictions derived from the present hierarchical gating model. The framework should not be interpreted as evidence for the model itself, but rather as one possible strategy for its empirical testing and potential falsification.

The design follows a factorial 2 × 2 randomized controlled structure that dissociates bottom-up (peripheral-to-spinal/brainstem) and top-down (cortical-to-subcortical) intervention dimensions, while also including a combined condition to examine potential additive or interaction effects. Participants undergo baseline multimodal assessment prior to randomization into four intervention conditions: control, bottom-up intervention, top-down intervention, and combined intervention. Bottom-up interventions are designed to target sensory gain, postural tone, and subcortical regulatory mechanisms through structured sensorimotor activity, whereas top-down interventions emphasize executive control, motor learning, attentional regulation, and context-dependent suppression of reflex-associated motor output.

The primary outcome is the change in primitive reflex persistence, quantified as a composite score derived from standardized assessments. Secondary outcomes include multimodal indices of hierarchical inhibitory regulation, including task-evoked cortical activation (e.g., fNIRS), intracortical inhibition measures (e.g., TMS-derived SICI/LICI), additional neurophysiological indices where applicable, and behavioral measures of sensorimotor and cognitive function. The framework further incorporates mechanistic mediation analyses designed to examine whether intervention-related neural changes covary with changes in primitive reflex expression and behavioral performance.

Importantly, the framework is specifically structured to permit mechanistic inference and potential falsification of the proposed model. The hierarchical gating hypothesis would be challenged if changes in primitive reflex expression occur independently of measurable changes in cortical or subcortical inhibitory indices, if bottom-up and top-down interventions fail to produce dissociable or additive effects, or if predicted relationships between neural and behavioral changes are not observed. Similarly, inconsistent findings across modalities or failure to replicate predicted associations would argue against the proposed mechanistic organization.

Accordingly, this figure should be interpreted as a theoretical–experimental research framework intended to guide longitudinal, multimodal, and interventional evaluation of the model, rather than as a validated mechanistic account or prescriptive intervention protocol.

### Distributed hierarchical gating of reflex expression

Primitive reflex suppression can be conceptualized as the emergent property of a multilayer hierarchical gating system. At the spinal level, presynaptic inhibition regulates sensory gain and shapes reflex responsiveness. At the brainstem level, reticulospinal and vestibulospinal networks modulate global excitability and muscle tone. At the cortical level, predictive top-down control dynamically reweights reflex pathways according to behavioral context, consistent with broader principles of sensorimotor learning, adaptive motor prediction, and context-dependent motor control (Cote and Gossard, 2003; Moreno-Lopez et al., 2016; Rudomin and Schmidt, 1999; Seki et al., 2003; Wolpert et al., 2011). Within predictive processing frameworks, such top-down modulation can be interpreted as precision-weighting of sensorimotor prediction errors, whereby hierarchical inhibitory control regulates the gain of reflex pathways in accordance with contextual expectations.

Reflex expression can be conceptually approximated as a nonlinear function of sensory input, tonic excitability, and hierarchical inhibitory gain across sensorimotor levels. A simplified heuristic formulation might express reflex output (R) as:


R=f(S,T).(1−I)

Where S denotes sensory input, T tonic excitability, and I the integrated inhibitory influence across spinal, brainstem, and cortical levels. This expression is intended as a conceptual heuristic rather than a biophysically specified or empirically validated computational model. Within this framework, small fluctuations in inhibitory balance may produce disproportionate changes in observable reflex behavior, potentially contributing to the sensitivity of primitive reflexes to developmental state, injury, disease, and aging. This conceptualization accommodates graded developmental transitions rather than the abrupt disappearance of reflexes and is consistent with broader principles of distributed sensorimotor regulation (Cote and Gossard, 2003; Rudomin and Schmidt, 1999; Seki et al., 2003).

Importantly, this account also accommodates biomechanical and dynamic-systems explanations (e.g., changes in strength-to-weight ratio or arousal) as interacting determinants of observable reflex output, rather than as alternatives that invalidate inhibitory mechanisms (Thelen et al., 1984).

These factors should be considered as potential contributors to observed reflex expression, rather than being fully subsumed under inhibitory control mechanisms.

Within this framework, primitive reflexes serve as accessible probes of hierarchical gating. Because they depend on coordinated inhibition across multiple levels, their expression provides a window into the integrity of distributed inhibitory networks.

### Lifespan symmetry of inhibitory decline

A striking symmetry emerges across the lifespan. Primitive reflexes fade as inhibitory systems mature and reappear as those systems degrade. Age-related decline in cortical GABA provides a neurochemical scaffold for this symmetry (Porges et al., 2021). Persistent reflexes in childhood predict adverse developmental outcomes, whereas re-emergent reflexes index frontal lobe dysfunction and dementia (Damasceno et al., 2005; Schott and Rossor, 2003).

In adults, the return of grasp, snout, and related frontal release signs reflects loss of corticobulbar inhibitory control and correlates with cognitive impairment. This symmetry reframes primitive reflexes as potential dynamic readouts of inhibitory architecture rather than developmental leftovers. They index the functional state of hierarchical gating mechanisms that are continuously reshaped across development and aging (Harp et al., 2022; Koller et al., 1982).

### Translational and methodological implications

Rigorous investigation requires objective multimodal measurement frameworks. Combining blinded behavioral ratings with fNIRS-based cortical measurements minimizes subjectivity and improves reliability (Futagi et al., 2012; Harp et al., 2022; Jensen et al., 1983).

Statistically, reflex expression can be modeled using hierarchical Bayesian frameworks that link behavioral indices to GABA concentration, cortical inhibition, and spinal gating. Latent variable models can estimate an underlying inhibitory construct from multimodal data, growth-curve models can characterize developmental trajectories, and machine-learning classifiers can evaluate predictive validity for clinical outcomes. Such approaches embed reflex assessment within contemporary computational neuroscience and precision medicine paradigms (Huys et al., 2016; Woo et al., 2017).

A particularly direct test of causality is a factorial 2 × 2 randomized controlled trial that independently manipulates bottom-up and top-down practice (and their combination) against an active control condition, with assessor blinding for reflex ratings and preregistered primary outcomes. Where feasible, multimodal inhibition proxies (e.g., TMS-derived cortical inhibition indices, MRS-based neurochemistry, and mobile functional neuroimaging during ecologically relevant tasks) can link reflex changes to changes in inhibitory architecture across levels.

### Open questions and experimental agenda

A central unresolved question concerns the causal relationship between movement patterns and reflex inhibition. Specifically, it remains to be determined whether repeated execution of primitive reflex motor patterns, or voluntary movements performed in opposition to those patterns, can suppress persistent reflexes through activity-dependent cortical stimulation and strengthening of inhibitory networks.

Emerging empirical work has begun to explore these mechanisms (Stephens-Sarlós et al., 2024a,b, 2025). In parallel, ongoing work is using functional near-infrared spectroscopy (fNIRS) to examine the relationship between persistent grasp and Babkin reflexes and cortical activation patterns. These experiments provide an opportunity to directly link reflex behavior with cortical dynamics and to test whether targeted sensorimotor protocols can measurably alter inhibitory architecture.

The school-age intervention evidence includes an institutional evaluation of the Sensorimotor Training Program, and the older-adult evidence includes a 16-week experimental investigation (Stephens-Sarlós et al., 2024a,b, 2025).

In line with non-primary article constraints, ongoing neuroimaging and protocol-development efforts are mentioned here only as measurement platforms; no unpublished primary results are reported.

This framework generates explicit, falsifiable predictions that can be operationalized using multimodal and large-scale empirical approaches. Specifically, the level of primitive reflex expression is expected to covary with quantitative indices of inhibitory control across the lifespan. For example, individuals with higher composite reflex scores are predicted to exhibit reduced task-evoked cortical activation in sensorimotor regions (e.g., BA4/BA6) during voluntary and reflex-related motor tasks, as measured by fNIRS. In neurodegenerative populations, the re-emergence of primitive reflexes is expected to be associated with decreased cortical activation and altered functional lateralization, consistent with reduced cortical inhibitory control.

Across development, primitive reflex profiles are hypothesized to predict variance in sensorimotor integration, attention, and related cognitive functions beyond age-related effects in large pediatric cohorts.

Furthermore, targeted sensorimotor interventions are predicted to induce concurrent reductions in primitive reflex persistence and measurable changes in cortical activation patterns, consistent with shifts in hierarchical inhibitory regulation. Testing these predictions will help determine whether primitive reflexes can serve as quantitative, mechanistically informative readouts and intervention targets within a systems-level model of brain development and decline. Importantly, these predictions are directly testable within ongoing neuroimaging, large-cohort, and intervention-based research frameworks.

A particularly direct mechanistic test is that individuals with higher primitive reflex composite scores should show reduced intracortical inhibition (e.g., lower TMS-derived SICI values), providing a measurable link between behavioral reflex indices and cortical inhibitory physiology.

A concrete developmental cascade prediction is that early differences in muscle tone and reflex pattern quality will prospectively relate to proxies of activity-dependent plasticity (e.g., peripheral BDNF, age-appropriate inhibition indices, and white matter maturation), which in turn will predict the emergence of context-dependent voluntary control (testable via preregistered mediation models) (Arichi et al., 2010; Fields, 2015; Huang et al., 1999; Szuhany et al., 2015).

## Discussion

This perspective proposes a systems-level framework in which primitive reflexes are conceptualized as potential quantitative behavioral markers of hierarchical inhibitory control across spinal, brainstem, and cortical systems. Rather than simply disappearing during development, primitive reflexes become progressively attenuated through processes of integration and inhibitory control as distributed sensorimotor systems mature.

A key implication is the possibility of a lifespan symmetry: reflex attenuation during development and reflex re-emergence in aging or neurological disease may arise from partially shared inhibitory dynamics.

This interpretation provides a potentially parsimonious link between developmental immaturity and neurodegenerative decline, connecting behavioral observations with underlying neurochemical and systems-level processes.

However, primitive reflex expression is not specific to inhibitory control and may also be influenced by multiple interacting factors, including muscle tone, postural control, arousal, sensory processing, attention, task comprehension, biomechanical constraints, and examiner-dependent scoring. Accordingly, reflex measures are unlikely to represent process-specific indices and should not be interpreted as uniquely indexing inhibitory control. Accordingly, the inhibitory-control framework should be interpreted as one explanatory model among several, rather than as a definitive mechanism.

The framework also highlights the potential translational value of primitive reflexes as accessible, low-cost indicators of inhibitory function. However, this potential depends on rigorous validation through standardized assessment, multimodal measurement, and experimentally controlled designs.

Importantly, the model is hypothesis-generating and requires empirical testing, particularly regarding causal relationships between movement, inhibitory maturation, and reflex modulation.

Several important limitations should be acknowledged. First, direct contemporary primary evidence linking prematurity, hypotonia, or hypertonia to qualitative variation in primitive reflex expression remains relatively limited and heterogeneous. Second, although converging evidence suggests that physical activity can modulate inhibitory neurobiology (e.g., GABAergic function, neurotrophic signaling, and activity-dependent plasticity), direct human evidence demonstrating that such changes causally reduce persistent primitive reflex expression is currently lacking. Third, frontal release signs are widely interpreted as indicators of reduced frontal inhibitory regulation, yet direct evidence that they specifically index corticobulbar inhibitory dysfunction remains incomplete. Fourth, multimodal studies directly linking primitive reflex expression with combined MRS-, TMS-, or other neurophysiological indices of inhibitory control are still sparse.

Finally, although the present framework emphasizes quantitative reflex profiling, primitive reflex assessment has historically been limited by substantial methodological heterogeneity, examiner dependence, and the absence of standardized scoring systems. To address this limitation, we developed a standardized assessment protocol covering 18 primitive and transitional reflexes with predefined elicitation and scoring criteria and preliminary evidence of high inter-rater and intra-rater reliability. Nevertheless, broader psychometric evaluation, external replication, construct validation, and independent cross-laboratory validation remain necessary before primitive reflex profiling can be considered a fully established candidate quantitative marker approach.

Importantly, these gaps should not be interpreted as contradictions of the present framework, but rather as critical empirical targets. Ongoing work within our broader research program is specifically designed to test, refine, and, where necessary, falsify these proposed relationships using longitudinal, neuroimaging, and intervention-based designs.

The proposed 2 × 2 randomized trial design should be interpreted as a future experimental framework intended to test, rather than support, the present model.

Accordingly, we do not claim that primitive reflex attenuation is currently proven to result from a single causal pathway involving GABAergic maturation, BDNF signaling, activity-dependent myelination, and cortical inhibition. Rather, we propose these mechanisms as biologically plausible and partially supported components of a testable systems-level framework. Future studies should determine which components are necessary, sufficient, or merely associated with primitive reflex modulation.

Future research should prioritize longitudinal, multimodal, and interventional approaches to determine whether primitive reflexes can serve as reliable quantitative behavioral readouts and mechanistically informative probes of distributed inhibitory control across the lifespan and, only after formal validation, candidate biomarkers. Such work will be essential for clarifying the specificity, sensitivity, and causal relevance of primitive reflex expression in relation to inhibitory neurobiology, cortical dynamics, and functional behavioral outcomes. Ultimately, the validity of the present framework will depend on whether these proposed relationships can be consistently replicated across developmental, aging, and clinical populations using rigorously controlled and mechanistically informative experimental designs.

## Data Availability

The original contributions presented in the study are included in the article/supplementary material, further inquiries can be directed to the corresponding author.
